# Supplementary material for: Imaging nodal knots in momentum space through topolectrical circuits
Source: Nat Commun. 2020 Sep 1;11:4385. doi: 10.1038/s41467-020-17716-1 (PMC7463261; doi:10.1038/s41467-020-17716-1)
Supplement: Supplementary file 1 — Supplementary Information [file 41467_2020_17716_MOESM1_ESM.pdf]

Supplementary Information

Imaging nodal knots in momentum space through topolectrical circuits

By Lee *et. al.*

I. SUPPLEMENTARY INFORMATION

Supplementary Note 1: Relation between drumhead states and surface topological band structure

Here we briefly illustrate how topological drumhead regions can be read from the surface band structure. Consider for instance a trefoil nodal knot, as shown in the left panels of Fig. 1. Drumhead regions are points in the surface Brillouin zone where topological zero modes exists. As evident in the surface band structures (Right panels) plotted along the dashed paths on the left, these zero modes must necessarily terminate at bulk gap closures, i.e. nodal lines. As such, drumhead states are necessarily demarcated by the surface nodal lines.

Supplementary Note 2: Alexander polynomial from the braid

The Alexander polynomial invariant of a knot can in fact be directly computed from its braid closure. At first sight, this seems tricky, because the closure of a series of braid operations do not uniquely define a knot/link, which can easily be topologically equivalent to a seemingly different braid. That said, there exists a direct means of obtaining the Alexander polynomial $A(t)$ via the (unreduced) Burau representation of a braid:

$$\sigma_i \to \sigma_i(t) = \mathbb{I}_{i-1} \oplus \begin{pmatrix} 1-t & t \\ 1 & 0 \end{pmatrix} \oplus \mathbb{I}_{N-i-1}, \tag{1}$$

where N is the total number of strands. A generic braid can be expressed as a composition of braid operations $\sigma^{\pm}_{i_1}\sigma^{\pm}_{i_2}\sigma^{\pm}_{i_3}...$, with corresponding Burau representation matrix $\sigma(t) = \sigma^{\pm}_{i_1}(t)\sigma^{\pm}_{i_2}(t)\sigma^{\pm}_{i_3}(t)....$ It turns out that the Alexander polynomial invariant is simply given by

$$A(t) = \det\left([\mathbb{I}_N - \sigma]_{11}(t),\right) \tag{2}$$

where $[\mathbb{I}_N - \sigma]_{11}(t)$ is the minor matrix of $\mathbb{I}_N - \sigma(t)$, which is obtained by omitting its first row and column. Note that when $t = 1$, $\sigma(1)$ just gives the permutation matrix for the entire braid, and that each independent permutation cycle gives rise to a separate line node. It is also conventional to normalize $A(t)$ by a power of t, such that it becomes symmetric in t and t^{-1}.

Supplementary Note 3: Further details of circuit simulation and implementation

Large-scale *Xyce* simulations provide a platform towards a realistic experimental setting of our circuit design. The compatibility of simulation and experiment for electrical circuits reaches an unprecedented degree of accuracy and agreement in comparison to any other architecture in which topological bands can unfold. A rigorous simulation includes the use of realistic voltage sources supplemented by corresponding shunt resistances, serving as the external excitation, i.e., inhomogeneities to the circuit's differential equations. The simulation further incorporates a realistic measurement process comprising a read-out of voltages at the circuit nodes and of the input current through the shunt resistance, which is analogous to an experimental framework. There, Lock-In amplifiers can be used for the corresponding measurements at fixed frequency. Similar to the experimental sequence of data analysis, the simulation data is subsequently post-processed to reconstruct the admittance band structure from global single-point voltage measurements. In principle, circuit simulations also allow to incorporate component tolerances and serial resistances to study disorder and parasitic effects.

Supplementary Note 4: Explicit forms of Nodal Knots

We next present the explicit forms of the nodal knots used in the above simulations. These may be appropriately truncated for experimental implementations, as is done for the Hopf-link experiment described later. The coefficients in the defining functions $z(\mathbf{k})$ and $w(\mathbf{k})$, which determine the precise nodal line can be modified as long as the winding number, which defines the mapping to the three dimensional Brillouin zone stays invariant. This implies, that small changes of the coefficients will only modify the shape and not the principal structure of the knot. Concerning the simulations of the knot structures, we fine-tuned those coefficients to an optimal value, that mediates between the complexity of *Xyce* simulations and the resolution of the knot in the three-dimensional momentum grid determined by the implemented system sizes.

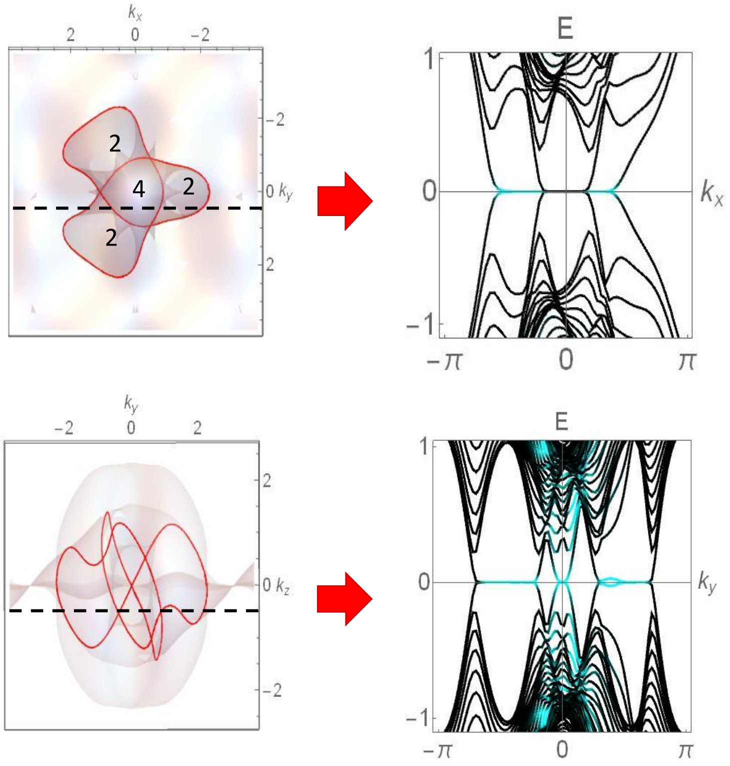

Supplementary Figure 1: Surface Trefoil knot nodal structure and drumhead states labeled by their multiplicities (Left panels), with corresponding surface band structures along the indicated dashed lines shown on the right. Blue/black curves indicated surface/bulk localization. The top and bottom panels illustrate the same system viewed from different angles.

1. Hopf-link circuit

For the Hopf-link, we employ a knot function of the form

$$f(z,w) = z^2 - w^2 \tag{3}$$

with $z(k_x,k_y,k_z)$ and $w(k_x,k_y,k_z)$ as defined in equation 7 of the main text. The real and imaginary part of $f(k_x,k_y,k_z)$, truncated to admit only nearest-neighbor couplings in each direction with commensurate magnitudes, are given by

$$\begin{aligned}\mathfrak{Re}\, f(k_x,k_y,k_z) =& -10 + 8\cos(k_x) - 2\cos(k_x-k_y) + 8\cos(k_y) - 2\cos(2k_y) - 2\cos(k_x+k_y) - 2\cos(k_x-k_z)\\ &- 2\cos(k_y-k_z) + 8\cos(k_z) + 2\cos(4k_z) - 2\cos(k_x+k_z) - 2\cos(k_y+k_z)\end{aligned} \tag{4a}$$

$$\begin{aligned}\mathfrak{Im}\, f(k_x,k_y,k_z) =& -2 + \cos(k_x) - \cos(k_x-k_y) + \cos(k_y) + \cos(k_x+k_y) + \cos(k_x-2k_z)\\ &+ \cos(k_y-2k_z) + 2\cos(k_z) - 4\cos(2k_z) + \cos(3k_z) + \cos(k_x+2k_z) + \cos(k_y+2k_z).\end{aligned} \tag{4b}$$

The corresponding circuit Laplacian is given by $J(k_x,k_y,k_z) = i\omega C[\mathfrak{Im}\, f(k_x,k_y,k_z)\tau_x + \mathfrak{Re}\, f(k_x,k_y,k_z)\tau_z]$, whose form in real space is illustrated in in Fig. 3, with LC normalized to unity.

2. Trefoil knot circuit

For the Trefoil knot, we employ a knot function of the form

$$f(z,w) = z^3 - w^2 \tag{5}$$

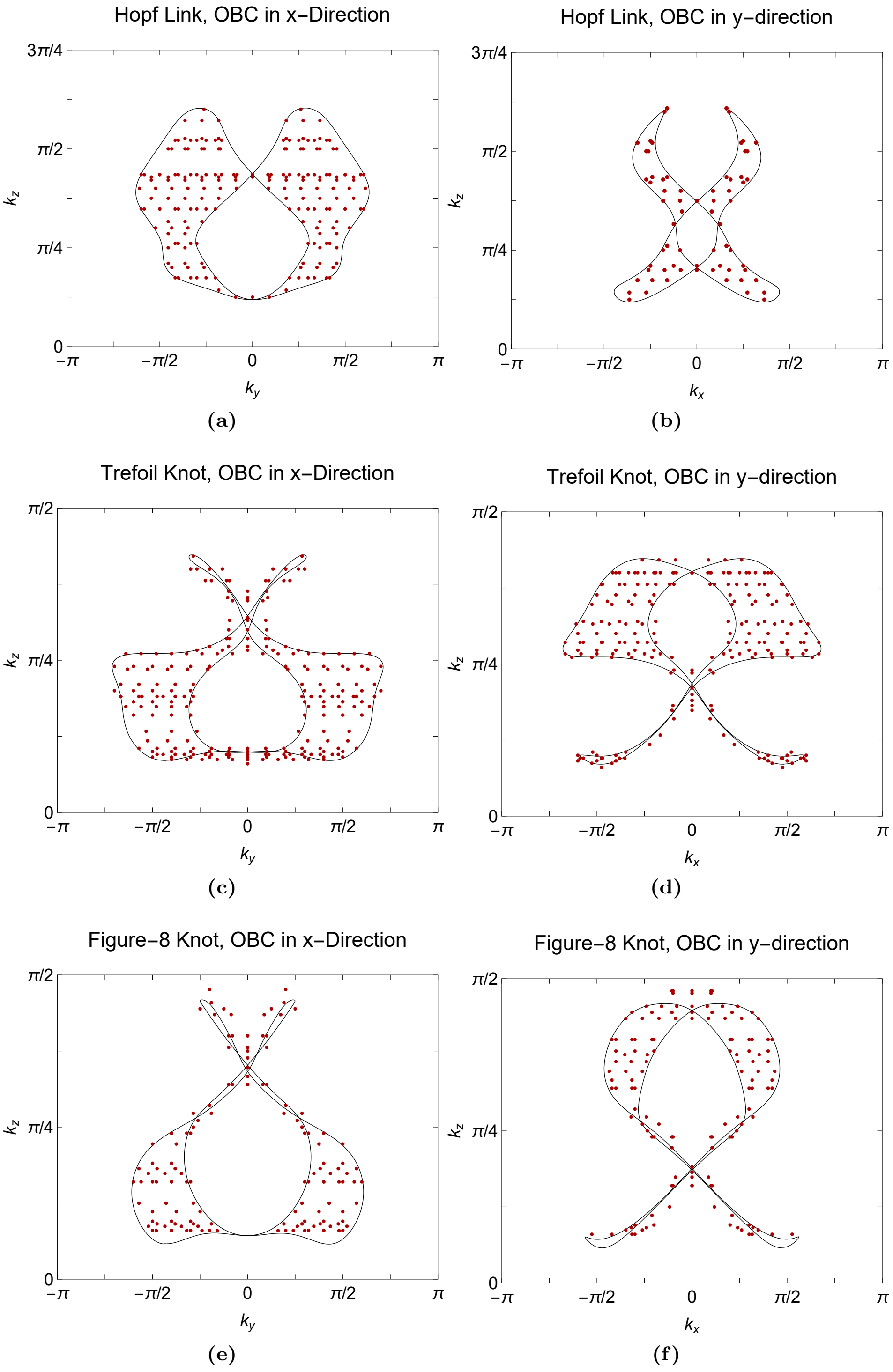

Supplementary Figure 2: Simulations of circuits implementing different nodal knot structures with OBCs in x- or y-direction as indicated. Red dots correspond to admittance eigenvalues, whose absolute value is smaller than an upper threshold of j_x or j_y for OBCs in x- or y-direction respectively. Black lines indicate the theoretical nodal knot projected onto the surface Brillouin zone. Hopf-links in (a) and (b) includes simulations with system sizes of $(22 \times 22 \times 16)$, $(23 \times 23 \times 20)$, $(16 \times 22 \times 19)$, $(22 \times 22 \times 14)$, $(25 \times 20 \times 23)$ and $(25 \times 24 \times 23)$, while $j_x = 0.0027\Omega^{-1}$ and $j_y = 0.0020\Omega^{-1}$. Trefoil knots in (c) and (d) includes simulations with system sizes of $(20 \times 20 \times 20)$, $(21 \times 21 \times 21)$, $(24 \times 15 \times 15)$, $(21 \times 20 \times 25)$, $(18 \times 19 \times 17)$, $(17 \times 18 \times 21)$, $(23 \times 21 \times 19)$, $(19 \times 25 \times 23)$ and $(20 \times 20 \times 22)$, while $j_x = 0.0030\Omega^{-1}$ and $j_y = 0.0025\Omega^{-1}$. Figure-8 knots in (e) and (f) includes simulations with system sizes of $(23 \times 23 \times 23)$, $(20 \times 20 \times 25)$, $(20 \times 20 \times 21)$, $(19 \times 16 \times 18)$, $(17 \times 14 \times 16)$, $(19 \times 25 \times 25)$ and $(25 \times 21 \times 22)$, while $j_x = 0.0028\Omega^{-1}$ and $j_y = 0.0032\Omega^{-1}$.

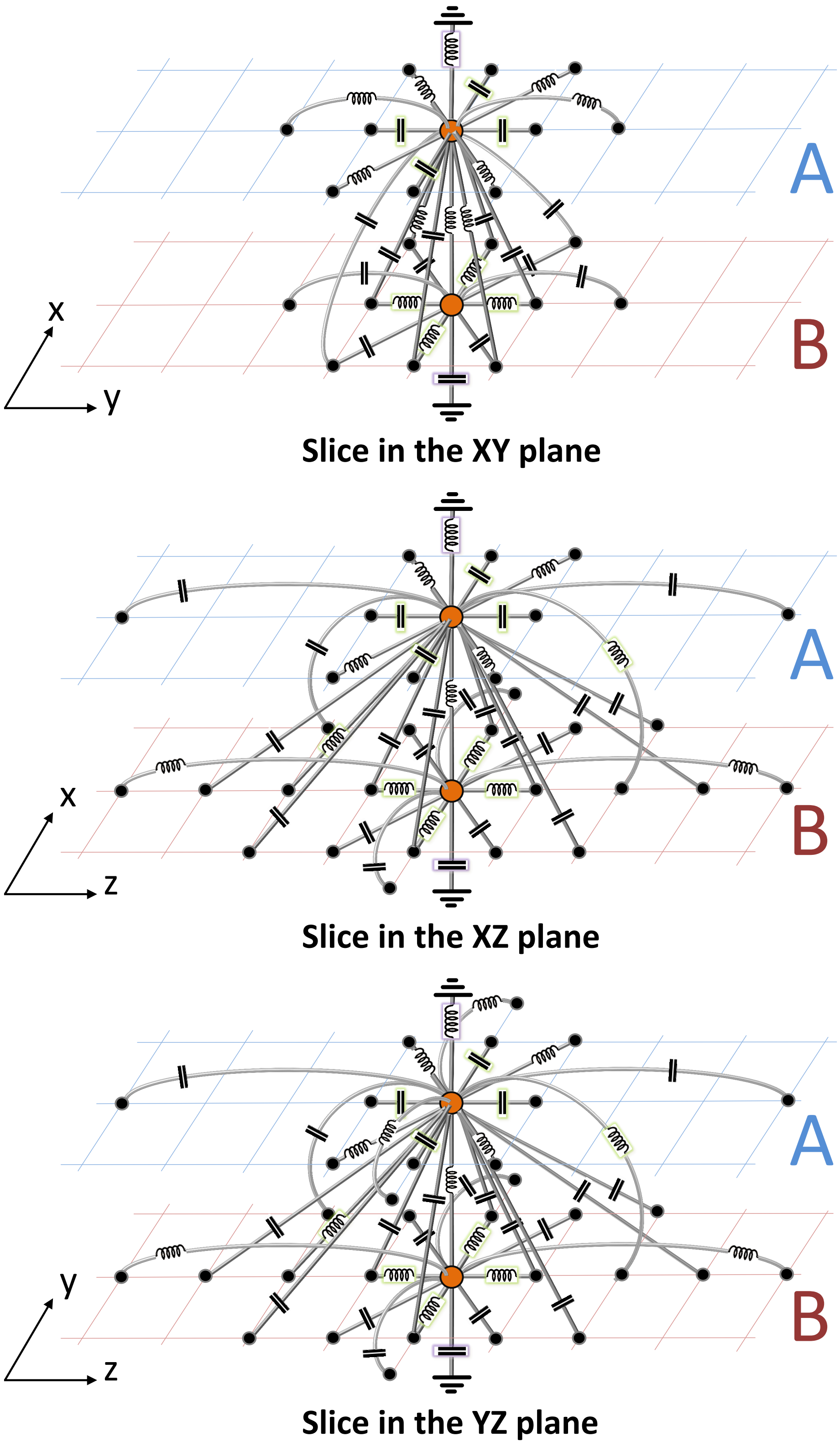

Supplementary Figure 3: Explicit illustration of the Hopf-link circuit in real-space. The circuit has an unit cell with sublattices A and B. For readability's sake, the 3D circuit network is expressed as slices in each of the 3 planes. For each slice, the connections are only shown emanating from one reference node (orange) from each sublattice. Also, for the inter-sublattice connections, only connections emanating from the reference node in A, and not B, are shown to avoid clutter. Each connection along x,y or z directions appear in two out of the three diagrams, while each connection in one of the planes appear only once. The black capacitors/inductors have magnitudes C and L, the purple glowing ones have magnitudes $2C$ and $\frac{L}{2}$, and the green glowing ones have magnitudes $4C$ and $\frac{L}{4}$.

with

$$z(k_x,k_y,k_z) = 1.15\cos(2k_z) + 0.1 + i(\cos(k_x) + \cos(k_y) + 1.25\cos(k_z) - 2.1), \tag{6a}$$
$$w(k_x,k_y,k_z) = \sin(k_x) + i\sin(k_y). \tag{6b}$$

The real and imaginary part of $f(k_x,k_y,k_z)$ with additonal truncations applied is then given by

$$\begin{aligned}\mathfrak{Re}\, f(k_x,k_y,k_z) =& -3.00566 + 1.26\cos(k_x) + 0.35\cos(2k_x) - 0.3\cos(k_x-k_y) + 1.26\cos(k_y)\\ &- 0.65\cos(2k_y) - 0.3\cos(k_x+k_y) - 2.15625\cos(k_x-3k_z) - 2.15625\cos(k_y-3k_z)\\ &+ 7.245\cos(k_x-2k_z) - 0.8625\cos(2k_x-2k_z) - 1.725\cos(k_x-k_y-2k_z)\\ &+ 7.245\cos(k_y-2k_z) - 1.725\cos(k_x+k_y-2k_z) - 0.8625\cos(2k_y-2k_z)\\ &- 2.53125\cos(k_x-k_z) - 2.53125\cos(k_y-k_z) + 10.6312\cos(k_z) - 20.419\cos(2k_z)\\ &+ 9.05625\cos(3k_z) - 1.14928\cos(4k_z) + 0.760438\cos(6k_z) - 2.53125\cos(k_x+k_z)\\ &- 2.53125\cos(k_y+k_z) + 7.245\cos(k_x+2k_z) - 0.8625\cos(2k_x+2k_z)\\ &- 1.725\cos(k_x-k_y+2k_z) + 7.245\cos(k_y+2k_z) - 1.725\cos(k_x+k_y+2k_z)\\ &- 0.8625\cos(2k_y+2k_z) - 2.15625\cos(k_x+3k_z) - 2.15625\cos(k_y+3k_z)\end{aligned} \tag{7a}$$

$$\begin{aligned}\mathfrak{Im}\, f(k_x,k_y,k_z) =& \ 16.254 - 15.81\cos(k_x) + 3.15\cos(2k_x) - 0.25\cos(3k_x) - 0.75\cos(k_x-2k_y) + 5.3\cos(k_x-k_y)\\ &- 0.75\cos(2k_x-k_y) - 15.81\cos(k_y) + 3.15\cos(2k_y) - 0.25\cos(3k_y) + 7.3\cos(k_x+k_y)\\ &- 0.75\cos(2k_x+k_y) - 0.75\cos(k_x+2k_y) + 0.991875\cos(k_x-4k_z) + 0.991875\cos(k_y-4k_z)\\ &- 0.826875\cos(k_x-2k_z) - 0.826875\cos(k_y-2k_z) + 7.875\cos(k_x-k_z) - 0.9375\cos(2k_x-k_z)\\ &- 1.875\cos(k_x-k_y-k_z) + 7.875\cos(k_y-k_z) - 1.875\cos(k_x+k_y-k_z) - 0.9375\cos(2k_y-k_z)\\ &- 18.8039\cos(k_z) + 3.47288\cos(2k_z) + 1.18281\cos(3k_z) - 4.16588\cos(4k_z)\\ &+ 1.23984\cos(5k_z) + 7.875\cos(k_x+k_z) - 0.9375\cos(2k_x+k_z) - 1.875\cos(k_x-k_y+k_z)\\ &+ 7.875\cos(k_y+k_z) - 1.875\cos(k_x+k_y+k_z) - 0.9375\cos(2k_y+k_z) - 0.826875\cos(k_x+2k_z)\\ &- 0.826875\cos(k_y+2k_z) + 0.991875\cos(k_x+4k_z) + 0.991875\cos(k_y+4k_z).\end{aligned} \tag{7b}$$

While the trigonometrical coefficients above are obtained from an exact expansions of $f(z,w)$, most of they can be varied about $20-30\%$ without changing the topology of the knot.

3. Figure-8 knot circuit

For the figure-8 knot, we employ a knot function of the form

$$\begin{aligned}f(z,w) = & \ 64\,z^3 - 12\,z\,(3 + 2(w^2 - \bar{w}^2))\\ & - 14\,(w^2+\bar{w}^2) - (w^4 - \bar{w}^4)\end{aligned} \tag{8}$$

with

$$z(k_x,k_y,k_z) = \cos(2k_z) + 0.1 + i(\cos(k_x) + \cos(k_y) + \cos(k_z) - 2), \tag{9a}$$
$$w(k_x,k_y,k_z) = \sin(k_x) + i\sin(k_y). \tag{9b}$$

The real and imaginary part of $f(k_x,k_y,k_z)$ with additional truncations applied is then given by

$$\begin{aligned}\mathfrak{Re}\, f(k_x,k_y,k_z) =& -147.536 + 76.8\cos(k_x) + 4.4\cos(2k_x) + 24\cos(k_x-2k_y) - 115.2\cos(k_x-k_y)\\ &+ 24\cos(2k_x-k_y) + 76.8\cos(k_y) - 23.6\cos(2k_y) + 76.8\cos(k_x+k_y) - 24\cos(2k_x+k_y)\\ &- 24\cos(k_x+2k_y) - 96\cos(k_x-3k_z) - 96\cos(k_y-3k_z) + 384\cos(k_x-2k_z) - 48\cos(2k_x-2k_z)\\ &- 96\cos(k_x-k_y-2k_z) + 384\cos(k_y-2k_z) - 96\cos(k_x+k_y-2k_z) - 48\cos(2k_y-2k_z)\\ &- 115.2\cos(k_x-k_z) + 24\cos(k_x-k_y-k_z) - 115.2\cos(k_y-k_z) - 24\cos(k_x+k_y-k_z)\\ &+ 460.8\cos(k_z) - 1051.68\cos(2k_z) + 384\cos(3k_z) - 38.4\cos(4k_z) + 16\cos(6k_z)\\ &- 115.2\cos(k_x+k_z) + 24\cos(k_x-k_y+k_z) - 115.2\cos(k_y+k_z) - 24\cos(k_x+k_y+k_z)\\ &+ 384\cos(k_x+2k_z) - 48\cos(2k_x+2k_z) - 96\cos(k_x-k_y+2k_z) + 384\cos(k_y+2k_z)\\ &- 96\cos(k_x+k_y+2k_z) - 48\cos(2k_y+2k_z) - 96\cos(k_x+3k_z) - 96\cos(k_y+3k_z) \end{aligned} \tag{10a}$$

and

$$\begin{aligned}\mathfrak{Im}\, f(k_x,k_y,k_z) =& 964.16 - 946.08\cos(k_x) + 192\cos(2k_x) - 16\cos(3k_x) - \cos(k_x-3k_y) - 48\cos(k_x-2k_y)\\ &+ 379.2\cos(k_x-k_y) - 48\cos(2k_x-k_y) + \cos(3k_x-k_y) - 946.08\cos(k_y) + 192\cos(2k_y)\\ &- 16\cos(3k_y) + 388.8\cos(k_x+k_y) - 48\cos(2k_x+k_y) - \cos(3k_x+k_y) - 48\cos(k_x+2k_y)\\ &+ \cos(k_x+3k_y) + 48\cos(k_x-4k_z) + 48\cos(k_y-4k_z) - 28.8\cos(k_x-2k_z)\\ &- 24\cos(k_x-k_y-2k_z) - 28.8\cos(k_y-2k_z) + 24\cos(k_x+k_y-2k_z) + 384\cos(k_x-k_z)\\ &- 48\cos(2k_x-k_z) - 96\cos(k_x-k_y-k_z) + 384\cos(k_y-k_z) - 96\cos(k_x+k_y-k_z)\\ &- 48\cos(2k_y-k_z) - 926.88\cos(k_z) + 115.2\cos(2k_z) + 51.2\cos(3k_z) - 192\cos(4k_z)\\ &+ 48\cos(5k_z) + 384\cos(k_x+k_z) - 48\cos(2k_x+k_z) - 96\cos(k_x-k_y+k_z) + 384\cos(k_y+k_z)\\ &- 96\cos(k_x+k_y+k_z) - 48\cos(2k_y+k_z) - 28.8\cos(k_x+2k_z) - 24\cos(k_x-k_y+2k_z)\\ &- 28.8\cos(k_y+2k_z) + 24\cos(k_x+k_y+2k_z) + 48\cos(k_x+4k_z) + 48\cos(k_y+4k_z). \end{aligned} \tag{10b}$$

Supplementary Note 5: Future Experimental Enhancements

In this experiment, we have developed a practical framework for constructing tunable circuit arrays that are sufficiently precise for reliably reproducing desired topological band structure features. A central challenge has been the tuning of repeated elements like inductors, which has to be accurately synchronized across all the repeating unit cells. We emphasize that this tunability has rarely been accomplished in the context of circuits with topological band structures.

Moving forward, the most crucial enhancement will be the automation of this tuning with a micro-controller. This will supersede our manual mechanical tuning of the variable inductors, which takes at least a minute for each component. Supplementary Figure 4 describes a solid-state automated tunable circuit with a MOSFET transistor controlling the wire loop around a fixed value inductor. By varying the gate voltage of the MOSFET through a micro-controller, the resistance in the wire loop may be electronically controlled to precisely vary the induced current in the wire loop and hence the impedance/inductance of the coupling unit. Requiring no manual control input i.e. visual reference to an oscilloscope, this approach can achieve drastic speedup for the tuning, and perhaps even allow real-time tuning for the simulation of Floquet Hamiltonians. While additional circuitry is required to connect all inductors to a common impedance measurement circuit, presenting an optimization challenge in dense complicated circuit arrays, this too can be streamlined with intelligent design approaches.

Another important advance will be the realization of nodal knots as a bona-fide spatial 3D circuit arrays. Nodal knot circuits possess more complicated structures than existing realizations of other 3D topolectrical circuits [1, 2], and will require more sophisticated PCB designs as well as a more extensive set of component i.e. capacitance values. To realize these capacitances as combinations of commercially available capacitors, it will be advantageous to use an artificial intelligence search-tree algorithm for finding the optimal combinations with fewest components or lowest cost. Related optimization algorithms can also be used to optimize the circuit array configuration in physical space, which is an important consideration when the circuit complexity increases [2, 3].

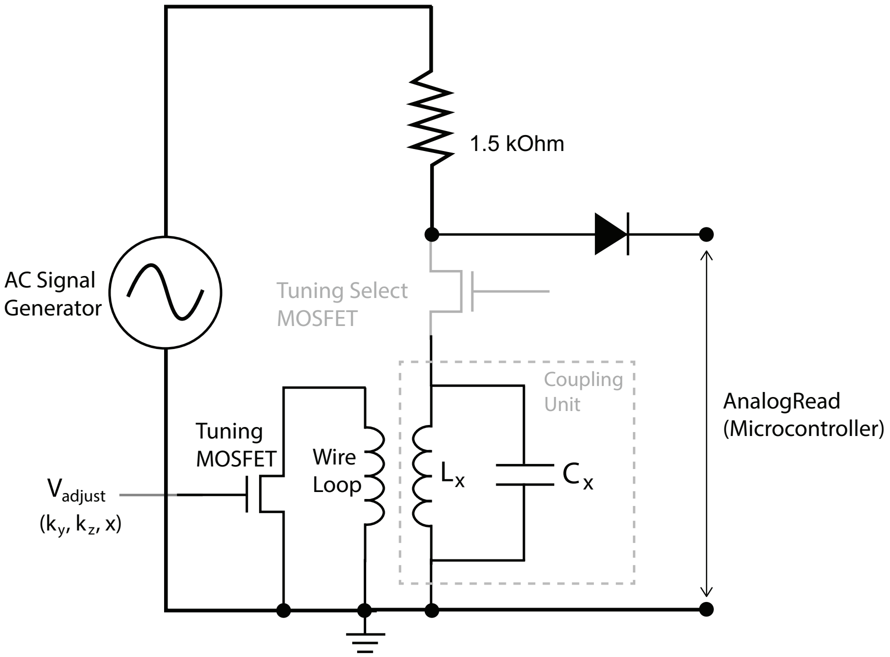

Supplementary Figure 4: Proposed circuit for automated tuning of variable inductors which make up the coupling units (logical components). The variable inductors consist of a wire loop surrounding fixed value inductors in series with a MOSFET transistor. The transistor controls the maximum current that may be induced in the wire loop, and therefore its effect on the magnetic field in the fixed value inductor and its resulting inductance. The micro-controller measures the amplitude of the AC potential across each coupling unit and adjusts the MOSFET voltage to achieve the desired impedance.

Supplementary Table 1: Capacitor values required in each logical component $t, -t, v, t_{AB}, g_A, g_B$. The exact required values from Eq. (27) in the Methods are slightly adapted to their target values possible with parallel combinations of standard commercially available capacitors. These parallel combinations should give a capacitance that agrees with its target capacitance values to at least 99.8%.

Component —	Exact (pF) —	Target (pF) —	Standard. Cap. Combi.(pF) —	Exp. Value
t	1755	1655	56,220,560,820	1656
v	1305	1205	56,330,820	1206
-t	3270	2770	10,560,2200	2770
tAB	3583	3083	10,56,820,2200	3086
gA	3655	3155	33,100,820,2200	3153
gB	4059	3559	33,220,3300	3553

Supplementary Table 2: List of physical components that make up the logical components in each unit cell, as labeled in the PCB diagram and photograph in Fig. 9. The fixed value inductor components have an uncertainty of +/-10% and a parasitic resistance of 0.10 Ω, while the fixed value capacitor components have an uncertainty of +/-5%.

Component	Value	Units	Manufacturer	Part Number
Lt, Lv	39	uH	Bourns JW Miller	RL622-390K-RC
Lmt, LtAB, LgA, LgB	10	uH	Bourns JW Miller	RL622-100K-RC
Ct0, Cv10, Cv20, CtAB1	56	pF	MULTICOMP PRO	MC0603N560J500CT
Ct1, CgB1	220	pF	WALSIN	0402N221J500CT
Ct2, Cmt1	560	pF	YAGEO	CC0805JRNPO9BN561
Ct3, Cv12, Cv22, CtAB2, CgA2	820	pF	WALSIN	0603N821J500CT
Cv11, Cv21	330	pF	WALSIN	0805N331J500CT
Cmt0, CtAB0	10	pF	MULTICOMP PRO	MC0603N100J500CT
Cmt2, CtAB3, CgA3	2200	pF	KEMET	C0402C222J5RACTU
CgA0, CgB0	33	pF	MULTICOMP PRO	MC0402N330J500CT
CgA1	100	pF	MURATA	GRM0335C1E101JA01D
CgB1	3300	pF	AVX	04025C332JAT2A

Supplementary Table 3: Impedance data at various sampling points (k_y, k_z) simulated at the theoretically determined AC frequency $f_0 = 795$ kHz, and measured at both $f_0 = 795$ kHz and the calibration-corrected frequency $f_{\text{exp.}} = 740$ kHz. $Z_{\text{i}}(\text{sim}, f_0)$ is the simulated impedance assuming ideal components. $Z_{\text{r}}(\text{sim}, f_0)$ is the simulated impedance when the empirically estimated tolerance of 1% and serial parasitic resistance of 0.11Ω and 0.03Ω are added to each inductor and capacitor, with $\Delta Z_{\text{r}}(\text{sim}, f_0)$ being the corresponding standard deviation in simulated impedance due to the 1% tolerance. $Z(\text{exp}, f_0)$ and $Z(\text{exp}, f_{\text{exp.}})$ are the experimentally measured impedance at $f_0 = 795$ kHz and $f_{\text{exp.}} = 740$ kHz respectively.

k_y	k_z	$Z_{\text{i}}(\text{sim}, f_0)/\Omega$	$Z_{\text{r}}(\text{sim}, f_0)/\Omega$	$\Delta Z_{\text{r}}(\text{sim}, f_0)/\Omega$	$Z(\text{exp}, f_0)/\Omega$	$Z(\text{exp}, f_{\text{exp.}})/\Omega$
0.01	0.42	320	322.9	46.7	252.3	298.6
0.53	0.48	339.8	357.2	58.2	193.0	228.1
0.86	0.62	286.8	290.8	34.8	252.3	389.2
1.02	0.75	391.3	542.1	117	311.6	555.0
1.10	0.88	323.7	364.7	76.6	212.3	398.7
0.96	1.07	98.2	108.6	19.3	200.7	268.9
0.53	1.12	266.8	310	36.9	256.4	338.2
0.07	1.26	77906.9	347.2	87.5	208.4	365.7
0.08	0.86	130.8	132.8	5.2	351.9	112.9
0.27	0.92	139.6	142.6	6.9	311.6	137.6
0.28	0.71	149.1	153.2	6.8	458.0	152.0
0.90	1.28	322.4	77.1	30.8	216.2	172.2
1.24	0.35	75	69.9	6.9	65.8	112.9
0.71	0.14	123.4	120.8	5.0	109.4	62.5

Supplementary Table 4: Simulated data of impedance values for a Hopf Link circuit with N=9. Impedance values in each column correspond to impedance of points k_y, k_z within a k radius of 0.03 from a particular centered point k_{y0}, k_{z0}, in which $\sqrt{(k_y - k_{y0})^2 + (k_z - k_{z0})^2} < 0.03$. This table shows only part of the full dataset, the full dataset is distributed over Tables 4-14.

Z(k_{y0}=0.01 k_{z0}=0.42)/Ω	Z(k_{y0}=0.53 k_{z0}=0.48)/Ω	Z(k_{y0}=0.86 k_{z0}=0.62)/Ω	Z(k_{y0}=1.02 k_{z0}=0.75)/Ω	Z(k_{y0}=1.10 k_{z0}=0.88)/Ω	Z(k_{y0}=0.96 k_{z0}=1.07)/Ω	Z(k_{y0}=0.53 k_{z0}=1.12)/Ω
318.8	339.2	282.8	362.8	309	109.1	266.6
451.7	340.6	274.2	324.3	280.4	137.6	318.3
420.3	329.8	295.6	315.9	281.3	104.2	291.5
395.8	320.7	289	308.1	282.7	79.6	275.6
376.4	313.1	283.6	300.9	284.4	76.6	272.2
360.6	397	279.2	294.3	286.4	85.8	344.8
347.5	375.1	275.5	288.2	288.9	95.5	349.7
336.4	357.8	272.5	282.6	291.8	105.2	335.3
326.9	343.9	270	355.2	295.1	114.5	307.2
318.7	332.5	267.9	344.6	284.2	123.3	283.2
311.4	323	266	334.6	284.4	163.4	272.2
304.8	315	318	325.3	285.1	123.6	272.8
298.9	308.2	307.4	316.6	286.1	93.9	281.1
293.4	302.2	298.6	308.7	287.4	72.4	294.8
288.4	296.9	291.4	301.3	289.2	79.4	313
283.7	434.5	285.5	294.6	291.3	88.8	319.7
279.3	404.1	280.8	288.4	293.9	98.5	337.5
451.7	380.6	276.9	282.7	296.8	107.9	350.4
420.3	362.1	273.7	277.6	300.3	117	347.9
395.9	347.3	271.1	381.3	304.3	125.7	325.3
318.8	339.2	282.8	362.8	309	109.1	266.6
451.7	340.6	274.2	324.3	280.4	137.6	318.3
420.3	329.8	295.6	315.9	281.3	104.2	291.5
395.8	320.7	289	308.1	282.7	79.6	275.6
376.4	313.1	283.6	300.9	284.4	76.6	272.2
360.6	397	279.2	294.3	286.4	85.8	344.8
347.5	375.1	275.5	288.2	288.9	95.5	349.7
336.4	357.8	272.5	282.6	291.8	105.2	335.3
326.9	343.9	270	355.2	295.1	114.5	307.2
318.7	332.5	267.9	344.6	284.2	123.3	283.2
311.4	323	266	334.6	284.4	163.4	272.2
304.8	315	318	325.3	285.1	123.6	272.8
298.9	308.2	307.4	316.6	286.1	93.9	281.1
293.4	302.2	298.6	308.7	287.4	72.4	294.8
288.4	296.9	291.4	301.3	289.2	79.4	313
283.7	434.5	285.5	294.6	291.3	88.8	319.7

Supplementary Table 5: Simulated data of impedance values for a Hopf Link circuit with N=9. Impedance values in each column correspond to impedance of points k_y, k_z within a k radius of 0.03 from a particular centered point k_{y0}, k_{z0}, in which $\sqrt{(k_y - k_{y0})^2 + (k_z - k_{z0})^2} < 0.03$. This table shows only part of the full dataset, the full dataset is distributed over Tables 4-14.

Z(k_{y0}=0.01 k_{z0}=0.42)/Ω	Z(k_{y0}=0.53 k_{z0}=0.48)/Ω	Z(k_{y0}=0.86 k_{z0}=0.62)/Ω	Z(k_{y0}=1.02 k_{z0}=0.75)/Ω	Z(k_{y0}=1.10 k_{z0}=0.88)/Ω	Z(k_{y0}=0.96 k_{z0}=1.07)/Ω	Z(k_{y0}=0.53 k_{z0}=1.12)/Ω
279.3	404.1	280.8	288.4	293.9	98.5	337.5
451.7	380.6	276.9	282.7	296.8	107.9	350.4
420.3	362.1	273.7	277.6	300.3	117	347.9
395.9	347.3	271.1	381.3	304.3	125.7	325.3
376.4	335.3	268.9	369.1	308.9	134	296.1
360.6	325.4	267.1	357.3	289	258.9	276.6
347.5	317.1	265.4	346.2	288.9	194.3	270.6
336.4	310	337.3	335.7	289.2	146.7	274.6
327	303.8	323.1	326.1	289.8	111.3	285.2
318.7	298.3	311.4	317.2	290.8	84.9	300.6
311.4	293.4	301.8	309.1	292.2	73.4	320.6
304.8	288.9	294	301.7	294	82.3	345.7
298.9	488.6	287.6	294.8	296.2	91.8	310
293.4	444.7	282.4	288.6	298.9	101.3	328.2
288.4	411.7	278.3	282.9	302	110.6	345.4
283.7	386.4	274.9	277.6	305.6	119.4	353.5
279.3	366.7	272.2	410.9	309.7	127.9	341.9
275.1	351	270	397.9	314.5	136.1	313.3
451.9	338.3	268.1	384.6	319.9	144.1	286.1
420.4	327.9	266.5	371.5	293.9	231.3	272
396	319.2	265.1	359.1	293.8	174.2	270.5
376.5	311.9	363.6	347.4	294	132	277.4
360.7	305.5	344.4	336.7	294.7	100.4	290.1
347.6	299.9	328.6	326.8	295.7	77	307.2
336.5	294.8	315.8	317.7	297.1	76.1	329
327	290.3	305.3	309.5	299	85.2	356.3
318.7	286	296.7	301.9	301.3	94.7	391.4
311.4	570.3	289.8	295.1	304	104	318.2
304.8	503.9	284.2	288.8	307.2	113.1	337
298.9	455.8	279.7	283	311	121.7	352.2
293.5	420	276.2	277.7	315.3	130	352.9
288.4	392.7	273.3	440.4	320.2	138	331.8
283.7	371.5	271	429.6	325.9	146	300.8
279.3	354.9	269.2	416.3	332.3	153.9	278
275.1	341.5	267.6	401.8	299.4	274.9	269.3
452.1	330.5	266.2	387.4	298.9	207	271.7

Supplementary Table 6: Simulated data of impedance values for a Hopf Link circuit with N=9. Impedance values in each column correspond to impedance of points k_y, k_z within a k radius of 0.03 from a particular centered point k_{y0}, k_{z0}, in which $\sqrt{(k_y - k_{y0})^2 + (k_z - k_{z0})^2} < 0.03$. This table shows only part of the full dataset, the full dataset is distributed over Tables 4-14.

Z(k_{y0}=0.01 k_{z0}=0.42)/Ω	Z(k_{y0}=0.53 k_{z0}=0.48)/Ω	Z(k_{y0}=0.86 k_{z0}=0.62)/Ω	Z(k_{y0}=1.02 k_{z0}=0.75)/Ω	Z(k_{y0}=1.10 k_{z0}=0.88)/Ω	Z(k_{y0}=0.96 k_{z0}=1.07)/Ω	Z(k_{y0}=0.53 k_{z0}=1.12)/Ω
420.6	321.4	264.9	373.5	298.7	156.6	281.2
396.1	313.8	373.5	360.5	298.9	119.1	295.6
376.6	307.2	352.1	348.4	299.6	90.8	314.5
360.8	301.4	334.8	337.4	300.6	70.4	338.1
347.6	296.3	320.6	327.3	302.1	78.9	368.1
336.6	291.6	309.1	318.2	304	88.1	406.7
327.1	287.3	299.7	309.8	306.3	97.5	458.2
318.8	594.6	292.1	302.2	309.2	106.7	308.4
311.5	520.9	286.1	295.3	312.5	115.5	327
304.9	467.9	281.2	288.9	316.4	123.9	345.6
298.9	428.9	277.4	283.2	320.9	132	356.5
293.5	399.5	274.4	458	326.1	139.9	347.5
288.5	376.8	272.1	449.2	331.9	147.8	319
283.7	359	270.2	436.1	338.6	155.6	289.2
279.3	344.8	268.6	420.8	346.3	163.4	272.1
275.1	333.3	267.3	405	304.5	246.2	268.4
452.5	323.8	266.1	389.6	303.8	185.9	274.1
420.9	315.8	384.5	375	303.6	141.2	285.7
396.3	309	360.7	361.5	303.8	107.6	301.9
376.8	303	341.5	349.2	304.5	82.4	322.4
360.9	297.7	325.9	337.9	305.5	72.9	348.2
347.8	293	313.2	327.7	307	81.6	381
336.7	288.6	302.9	318.5	309	90.9	423.8
327.2	284.6	294.7	310.1	311.4	100.2	481.2
318.9	621.8	288.1	302.4	314.4	109.1	316.8
311.5	539.6	282.8	295.5	317.9	117.7	336.2
305	481.3	278.7	289.1	321.9	125.9	353.3
299	438.7	275.5	283.4	326.6	133.9	356.9
293.5	406.8	273.1	473.1	332	141.7	337.4
288.5	382.4	271.1	468.5	338.1	149.5	305.1
283.8	363.4	269.6	456.9	345.1	157.2	279.5
279.4	348.3	268.3	441.5	353.1	165	268.3
452.9	336.2	267.2	424.5	309.5	220.9	269
421.2	326.2	266.1	407.5	308.9	167.4	277.4
396.6	317.9	396.8	391.3	308.6	127.6	291
377	310.8	370.3	376.1	308.8	97.4	308.8

Supplementary Table 7: Simulated data of impedance values for a Hopf Link circuit with N=9. Impedance values in each column correspond to impedance of points k_y, k_z within a k radius of 0.03 from a particular centered point k_{y0}, k_{z0}, in which $\sqrt{(k_y - k_{y0})^2 + (k_z - k_{z0})^2} < 0.03$. This table shows only part of the full dataset, the full dataset is distributed over Tables 4-14.

Z(k_{y0}=0.01 k_{z0}=0.42)/Ω	Z(k_{y0}=0.53 k_{z0}=0.48)/Ω	Z(k_{y0}=0.86 k_{z0}=0.62)/Ω	Z(k_{y0}=1.02 k_{z0}=0.75)/Ω	Z(k_{y0}=1.10 k_{z0}=0.88)/Ω	Z(k_{y0}=0.96 k_{z0}=1.07)/Ω	Z(k_{y0}=0.53 k_{z0}=1.12)/Ω
361.1	304.7	348.9	362.3	309.4	75.1	331.2
347.9	299.3	331.7	349.7	310.5	75.5	359.3
336.8	294.4	317.7	338.3	312.1	84.4	395.4
327.3	290	306.5	328	314.1	93.6	442.9
319	285.8	297.4	318.7	316.6	102.7	507.1
311.6	652.4	290.2	310.3	319.6	111.5	325.8
305	560.5	284.5	302.6	323.3	119.9	345.5
299.1	496	280.1	295.7	327.5	127.9	358.6
293.6	449.3	276.6	289.4	332.3	135.8	352
288.6	414.7	274	483.2	338	143.5	323.6
283.9	388.4	272.1	485.7	344.4	151.1	292
279.4	368.1	270.5	477.9	351.7	158.8	272.2
421.7	352.1	269.3	463.5	360.1	166.5	266.6
397	339.2	268.3	445.9	314.6	198.7	270.9
377.3	328.7	267.3	427.4	313.9	151.2	281.6
361.4	320	410.5	409.3	313.6	115.5	296.9
348.1	312.7	380.8	392.4	313.8	88.4	316.4
337	306.4	357.1	376.9	314.4	69.7	340.8
327.4	300.8	338	362.8	315.5	78.1	371.6
319.1	295.8	322.7	350	317.1	87.1	411.4
311.7	291.3	310.3	338.5	319.2	96.2	464.4
305.1	287.1	300.4	328.2	321.8	105.2	536
299.2	687	292.5	318.9	324.9	113.7	335.4
293.7	583.8	286.3	310.5	328.7	121.9	353.9
288.7	512.2	281.4	302.9	333.1	129.8	359.8
283.9	461	277.8	296	338.2	137.5	341.5
397.4	423.3	275	485.2	344	145.1	308.5
377.6	394.9	272.9	498.2	350.7	152.7	280.8
361.6	373.1	271.4	497.2	358.4	160.3	267.4
348.4	356	270.3	485.9	367.1	167.9	266.6
337.2	342.4	269.3	468.8	319.7	179.3	273.9
327.6	331.4	268.5	449.2	318.9	136.9	286.6
319.3	322.3	425.8	429.4	318.6	104.8	303.6
311.9	314.7	392.5	410.5	318.8	80.5	324.8
305.3	308.1	366.1	393.1	319.4	72.1	351.4
299.3	302.4	345	377.2	320.5	80.7	385.2

Supplementary Table 8: Simulated data of impedance values for a Hopf Link circuit with N=9. Impedance values in each column correspond to impedance of points k_y, k_z within a k radius of 0.03 from a particular centered point k_{y0}, k_{z0}, in which $\sqrt{(k_y - k_{y0})^2 + (k_z - k_{z0})^2} < 0.03$. This table shows only part of the full dataset, the full dataset is distributed over Tables 4-14.

Z(k_{y0}=0.01 k_{z0}=0.42)/Ω	Z(k_{y0}=0.53 k_{z0}=0.48)/Ω	Z(k_{y0}=0.86 k_{z0}=0.62)/Ω	Z(k_{y0}=1.02 k_{z0}=0.75)/Ω	Z(k_{y0}=1.10 k_{z0}=0.88)/Ω	Z(k_{y0}=0.96 k_{z0}=1.07)/Ω	Z(k_{y0}=0.53 k_{z0}=1.12)/Ω
293.8	297.3	328.1	363	322.2	89.7	429.3
288.8	292.7	314.5	350.1	324.3	98.8	488.6
362	288.5	303.6	338.7	327	107.5	567.9
348.6	726.3	294.9	328.3	330.3	115.8	360
337.4	610	288.1	319.1	334.2	123.8	355.1
327.8	530.2	282.9	310.7	338.7	131.6	326.8
319.4	473.8	278.9	502.6	344	139.2	297.5
312	432.7	275.9	512.2	350.1	146.7	272.3
305.4	401.9	273.8	507.2	357.1	154.2	264.9
299.4	378.5	272.3	492.4	365.1	161.7	268
N/A	360.3	271.1	472.7	324	169.3	277.9
N/A	345.8	270.3	451.5	323.6	124.2	292.3
N/A	334.2	405.5	430.6	323.7	95.2	310.9
N/A	324.7	376.1	411.1	324.4	73.6	334
N/A	316.7	352.7	393.3	325.5	74.6	363
N/A	309.9	334	377.3	327.2	83.3	400.3
N/A	304	319.1	362.9	329.4	92.3	449.4
N/A	298.8	307.1	350.1	332.2	101.2	515.7
N/A	294.1	297.6	338.6	335.6	109.7	601.9
N/A	639.3	290.2	328.4	339.6	117.9	361.7
N/A	550.2	284.4	319.2	344.3	125.7	344
N/A	487.8	280.1	495.7	349.8	133.3	310.2
N/A	442.8	276.9	519.4	356.2	140.8	285.8
N/A	409.5	274.6	524.8	363.5	148.2	266.5
N/A	384.3	273	515.5	371.9	155.6	264.3
N/A	364.8	272	497.4	328.6	163.1	270.7
N/A	349.4	271.2	475.4	328.7	86.7	282.6
N/A	337.1	387.1	452.7	329.3	68.9	298.7
N/A	327.1	361.2	431	330.5	77.1	319
N/A	318.8	340.5	411	332.2	85.9	344.2
N/A	311.8	324.1	393.1	334.5	94.8	375.9
N/A	305.7	310.9	377	337.4	103.5	417.1
N/A	300.4	300.5	362.7	340.9	111.8	472.1
N/A	295.6	292.3	349.9	345.1	119.7	545.9
N/A	572.4	286	338.6	350	127.4	330.3
N/A	503.3	281.3	515.4	355.7	134.9	304.6

Supplementary Table 9: Simulated data of impedance values for a Hopf Link circuit with N=9. Impedance values in each column correspond to impedance of points k_y, k_z within a k radius of 0.03 from a particular centered point k_{y0}, k_{z0}, in which $\sqrt{(k_y - k_{y0})^2 + (k_z - k_{z0})^2} < 0.03$. This table shows only part of the full dataset, the full dataset is distributed over Tables 4-14.

Z(k_{y0}=0.01 k_{z0}=0.42)/Ω	Z(k_{y0}=0.53 k_{z0}=0.48)/Ω	Z(k_{y0}=0.86 k_{z0}=0.62)/Ω	Z(k_{y0}=1.02 k_{z0}=0.75)/Ω	Z(k_{y0}=1.10 k_{z0}=0.88)/Ω	Z(k_{y0}=0.96 k_{z0}=1.07)/Ω	Z(k_{y0}=0.53 k_{z0}=1.12)/Ω
N/A	453.9	277.8	535.3	362.3	142.3	271.9
N/A	417.7	275.4	535.7	369.9	149.6	263.2
N/A	390.5	273.8	522	333.6	157	265.2
N/A	369.6	272.7	500.8	334.2	71.2	274.3
N/A	353.2	370.5	476.8	335.4	79.6	288.1
N/A	340.2	347.7	453	337.2	88.4	305.8
N/A	329.7	329.6	430.7	339.5	97.2	327.8
N/A	321	315.1	410.5	342.5	105.7	355.3
N/A	313.7	303.7	392.5	346.1	113.8	390.2
N/A	307.5	294.7	376.5	350.5	121.5	436
N/A	302	287.8	362.2	355.6	129.1	497.5
N/A	466.1	282.6	549.5	361.5	136.4	265.2
N/A	426.6	278.8	544.5	368.4	143.7	262
N/A	397.2	276.1	526.5	340.2	151	267.5
N/A	374.7	319.7	502.5	342.1	90.8	278.8
N/A	357.3	307.1	476.9	344.5	99.5	294.2
N/A	343.5	297.3	452.3	347.6	107.7	313.6
N/A	332.4	289.6	429.8	351.3	115.6	337.5
N/A	323.3	283.9	409.5	355.8	123.2	367.5
N/A	315.7	N/A	N/A	361.1	130.6	406.1
N/A	380.2	N/A	N/A	N/A	137.9	284.1
N/A	361.6	N/A	N/A	N/A	N/A	301.1
N/A	346.9	N/A	N/A	N/A	N/A	322.1

Supplementary Table 10: Simulated data of impedance values for a Hopf Link circuit with N=9. Impedance values in each column correspond to impedance of points k_y, k_z within a k radius of 0.03 from a particular centered point k_{y0}, k_{z0}, in which $\sqrt{(k_y - k_{y0})^2 + (k_z - k_{z0})^2} < 0.03$. This table shows only part of the full dataset, the full dataset is distributed over Tables 4-14.

Z(k_{y0}=0.07 k_{z0}=1.26)/Ω	Z(k_{y0}=0.08 k_{z0}=0.86)/Ω	Z(k_{y0}=0.27 k_{z0}=0.92)/Ω	Z(k_{y0}=0.28 k_{z0}=0.71)/Ω	Z(k_{y0}=0.9 k_{z0}=1.28)/Ω	Z(k_{y0}=1.24 k_{z0}=0.35)/Ω	Z(k_{y0}=0.71 k_{z0}=0.14)/Ω
259.1	130.8	139.4	148.7	77.9	75.1	123.3
373.5	130.4	137.4	150.2	94	80.4	125.3
277.1	130.5	137.8	149.4	85.1	80.1	125.8
234.4	130.5	138.1	148.7	78.2	79.9	126.3
692.7	130.5	138.4	148	73.9	79.6	126.9
630.3	130.6	138.8	147.3	127.6	79.3	127.5
508.5	130.6	139.2	146.6	114.7	79	123.4
368.9	130.7	139.6	146	103.3	78.7	123.9
274.5	130.8	140	152	93.2	78.3	124.4
233.6	130.9	137.1	151.2	84.4	80.1	124.9
222.5	130.5	137.3	150.4	78.1	79.9	125.4
217.1	130.5	137.6	149.6	73.8	79.7	125.9
217.7	130.5	137.9	148.9	70.1	79.4	126.5
678.6	130.5	138.3	148.2	66.9	79.2	127
712.1	130.6	138.6	147.5	64.3	78.9	127.6
691.1	130.6	139	146.9	140.5	78.6	128.2
627.2	130.7	139.4	146.2	126.4	78.3	122.6
502.9	130.8	139.8	145.6	113.7	78	123
363.8	130.8	140.2	145	102.4	77.6	123.5
271.8	131	140.6	153	92.4	77.2	124
232.8	131.1	141.1	152.2	83.8	76.8	124.5
222	131.2	137	151.4	78	79.7	125
216.9	130.6	137.2	150.6	73.7	79.5	125.5
217.7	130.5	137.5	149.9	70	79.2	126
223.3	130.5	137.8	149.1	66.8	79	126.6
232.2	130.5	138.1	148.4	64.2	78.8	127.1
597.9	130.6	138.5	147.7	62	78.5	127.7
682.1	130.6	138.8	147.1	60.3	78.2	128.3
711	130.6	139.2	146.4	154.7	77.9	128.9
689	130.7	139.6	145.8	139.3	77.6	121.7
623.6	130.8	140	145.2	125.3	77.2	122.2
496.7	130.9	140.4	144.6	112.7	76.9	122.6
358.4	131	140.8	144.1	101.6	76.5	123.1
268.9	131.1	141.3	154.1	91.8	76.1	123.5
231.9	131.3	141.8	153.3	83.3	75.7	124
221.4	131.4	136.9	152.4	77.9	79	124.5

Supplementary Table 11: Simulated data of impedance values for a Hopf Link circuit with N=9. Impedance values in each column correspond to impedance of points k_y, k_z within a k radius of 0.03 from a particular centered point k_{y0}, k_{z0}, in which $\sqrt{(k_y - k_{y0})^2 + (k_z - k_{z0})^2} < 0.03$. This table shows only part of the full dataset, the full dataset is distributed over Tables 4-14.

Z(k_{y0}=0.07 k_{z0}=1.26)/Ω	Z(k_{y0}=0.08 k_{z0}=0.86)/Ω	Z(k_{y0}=0.27 k_{z0}=0.92)/Ω	Z(k_{y0}=0.28 k_{z0}=0.71)/Ω	Z(k_{y0}=0.9 k_{z0}=1.28)/Ω	Z(k_{y0}=1.24 k_{z0}=0.35)/Ω	Z(k_{y0}=0.71 k_{z0}=0.14)/Ω
216.7	130.6	137.2	151.6	73.6	78.8	125
217.8	130.6	137.4	150.8	69.9	78.6	125.6
223.5	130.6	137.7	150.1	66.8	78.3	126.1
232.6	130.6	138	149.4	64.1	78.1	126.7
244	130.6	138.3	148.6	62	77.8	127.3
602.3	130.6	138.6	148	60.3	77.5	127.9
685.9	130.6	139	147.3	58.9	77.2	128.5
709.5	130.7	139.4	146.6	170.2	76.9	129.1
686.4	130.8	139.8	146	153.4	76.5	120.9
619.5	130.8	140.2	145.4	138.1	76.2	121.3
489.9	130.9	140.6	144.8	124.3	75.8	121.8
352.9	131	141	144.3	111.9	75.4	122.2
265.8	131.2	141.5	154.3	100.8	74.9	122.7
230.9	131.3	142	153.5	91.2	74.5	123.1
220.9	131.5	136.9	152.7	82.9	78.6	123.6
216.5	130.7	137.1	151.9	77.9	78.4	124.1
217.8	130.7	137.4	151.1	73.6	78.1	124.6
223.8	130.7	137.6	150.3	69.8	77.9	125.1
233	130.6	137.9	149.6	66.7	77.7	125.7
244.5	130.6	138.2	148.9	64	77.4	126.2
257.9	130.6	138.5	148.2	61.9	77.1	126.8
526.3	130.6	138.8	147.5	60.2	76.8	127.4
607.1	130.7	139.2	146.9	58.9	76.5	128
689.7	130.7	139.6	146.2	168.8	76.2	128.6
707.4	130.8	140	145.6	152.1	75.8	120.5
683.3	130.9	140.4	145.1	137	75.5	120.9
614.8	131	140.8	144.5	123.4	75.1	121.3
482.6	131.1	141.2	143.9	111.1	74.6	121.8
347.3	131.2	141.7	155.5	100.2	74.2	122.2
262.5	131.4	142.2	154.6	90.6	73.7	122.7
230	131.5	142.7	153.7	82.9	77.9	123.2
220.3	131.7	137.1	152.9	77.9	77.7	123.7
216.3	130.8	137.3	152.1	73.5	77.5	124.2
217.9	130.7	137.6	151.3	69.8	77.3	124.7
224.1	130.7	137.8	150.6	66.6	77	125.2
233.4	130.7	138.1	149.8	64	76.7	125.8
245.1	130.7	138.4	149.1	61.8	76.5	126.4

Supplementary Table 12: Simulated data of impedance values for a Hopf Link circuit with N=9. Impedance values in each column correspond to impedance of points k_y, k_z within a k radius of 0.03 from a particular centered point k_{y0}, k_{z0}, in which $\sqrt{(k_y - k_{y0})^2 + (k_z - k_{z0})^2} < 0.03$. This table shows only part of the full dataset, the full dataset is distributed over Tables 4-14.

Z(k_{y0}=0.07 k_{z0}=1.26)/Ω	Z(k_{y0}=0.08 k_{z0}=0.86)/Ω	Z(k_{y0}=0.27 k_{z0}=0.92)/Ω	Z(k_{y0}=0.28 k_{z0}=0.71)/Ω	Z(k_{y0}=0.9 k_{z0}=1.28)/Ω	Z(k_{y0}=1.24 k_{z0}=0.35)/Ω	Z(k_{y0}=0.71 k_{z0}=0.14)/Ω
258.6	130.7	138.7	148.4	60.1	76.2	127
530.5	130.7	139	147.8	58.8	75.8	127.6
612.3	130.7	139.4	147.1	57.8	75.5	128.2
693.7	130.8	139.8	146.5	167.5	75.1	128.8
704.7	130.9	140.2	145.9	151	74.7	120.1
679.6	130.9	140.6	145.3	136	74.3	120.5
609.4	131	141	144.7	122.5	73.9	120.9
474.7	131.1	141.4	144.2	110.4	73.5	121.4
341.3	131.3	141.9	155.7	99.6	73	121.8
259.1	131.4	142.4	154.9	90.1	72.5	122.3
228.9	131.6	142.9	154	82.9	77.3	122.8
219.7	131.7	137.3	153.2	77.9	77.1	123.2
216.1	130.8	137.5	152.4	73.5	76.8	123.8
218	130.8	137.8	151.6	69.8	76.6	124.3
224.4	130.8	138	150.8	66.6	76.3	124.8
233.9	130.7	138.3	150.1	64	76.1	125.4
245.7	130.7	138.6	149.4	61.8	75.8	125.9
259.3	130.7	138.9	148.7	60	75.5	126.5
535.1	130.8	139.2	148	58.7	75.1	127.1
617.8	130.8	139.6	147.3	57.7	74.8	127.7
697.7	130.8	140	146.7	166.3	74.4	128.3
701.2	130.9	140.4	146.1	150	74	119.7
675.1	131	140.8	145.5	135.2	73.6	120.1
603.3	131.1	141.2	144.9	121.8	73.2	120.5
466.2	131.2	141.7	144.4	109.7	72.7	121
334.9	131.3	142.1	156	99	72.3	121.4
255.5	131.5	142.6	155.1	89.7	71.8	121.9
227.9	131.6	143.1	154.3	82.9	76.6	122.3
219.1	131.8	137.5	153.4	77.9	76.4	122.8
215.9	130.9	137.7	152.6	73.6	76.2	123.3
218.2	130.9	138	151.8	69.8	76	123.8
224.8	130.8	138.2	151.1	66.6	75.7	124.4
234.5	130.8	138.5	150.3	63.9	75.4	124.9
246.4	130.8	138.8	149.6	61.7	75.1	125.5
260	130.8	139.1	148.9	60	74.8	126
540.1	130.8	139.5	148.2	58.7	74.5	126.6
623.8	130.8	139.8	147.6	57.7	74.1	127.2

Supplementary Table 13: Simulated data of impedance values for a Hopf Link circuit with N=9. Impedance values in each column correspond to impedance of points k_y, k_z within a k radius of 0.03 from a particular centered point k_{y0}, k_{z0}, in which $\sqrt{(k_y - k_{y0})^2 + (k_z - k_{z0})^2} < 0.03$. This table shows only part of the full dataset, the full dataset is distributed over Tables 4-14.

Z(k_{y0}=0.07 k_{z0}=1.26)/Ω	Z(k_{y0}=0.08 k_{z0}=0.86)/Ω	Z(k_{y0}=0.27 k_{z0}=0.92)/Ω	Z(k_{y0}=0.28 k_{z0}=0.71)/Ω	Z(k_{y0}=0.9 k_{z0}=1.28)/Ω	Z(k_{y0}=1.24 k_{z0}=0.35)/Ω	Z(k_{y0}=0.71 k_{z0}=0.14)/Ω
701.6	130.9	140.2	146.9	165.2	73.7	127.9
699.4	131	140.6	146.3	149	73.3	119.3
669.8	131.1	141	145.7	134.4	72.9	119.7
596.5	131.1	141.4	145.2	121.1	72.5	120.1
457.2	131.3	141.9	144.6	109.1	72	120.6
328.1	131.4	142.4	155.4	98.6	71.5	121
251.9	131.5	142.8	154.5	89.3	71	121.4
226.8	131.7	143.4	153.7	83	76	121.9
218.5	131.9	137.9	152.9	78	75.8	122.4
215.7	130.9	138.2	152.1	73.6	75.6	122.9
218.3	130.9	138.4	151.3	69.8	75.3	123.4
225.2	130.9	138.7	150.6	66.6	75	123.9
235	130.8	139	149.9	63.9	74.7	124.5
247.1	130.9	139.3	149.2	61.7	74.4	125
260.8	130.9	139.7	148.5	60	74.1	125.6
630.1	130.9	140	147.8	58.6	73.8	126.2
705.3	131	140.4	147.2	57.6	73.4	126.8
701.6	131	140.8	146.6	164.2	73	127.4
666.7	131.1	141.2	146	148.2	72.6	119.3
588.9	131.2	141.6	145.4	133.6	72.2	119.7
447.6	131.3	142.1	144.8	120.5	71.8	120.1
321	131.4	142.6	155.7	108.6	71.3	120.6
248.1	131.6	143.1	154.8	98.1	70.8	121
225.7	131.7	143.6	154	89	75.2	121.5
217.9	131.9	138.1	153.1	83.1	74.9	122
215.5	131	138.4	152.3	78.1	74.7	122.5
218.5	130.9	138.6	151.6	73.7	74.4	123
225.6	130.9	138.9	150.8	69.9	74.1	123.5
235.7	130.9	139.2	150.1	66.6	73.8	124
247.8	130.9	139.5	149.4	63.9	73.4	124.6
261.7	130.9	139.9	148.7	61.7	73.1	125.1
636.8	131	140.2	148.1	59.9	72.7	125.7
708.8	131	140.6	147.4	58.5	72.3	126.3
703.4	131.1	141	146.8	147.5	71.9	118.9
665.7	131.2	141.4	146.2	133	71.5	119.3
580.4	131.3	141.9	145.6	119.9	71.1	119.7
437.6	131.4	142.3	145.1	108.2	70.6	120.2

Supplementary Table 14: Simulated data of impedance values for a Hopf Link circuit with N=9. Impedance values in each column correspond to impedance of points k_y, k_z within a k radius of 0.03 from a particular centered point k_{y0}, k_{z0}, in which $\sqrt{(k_y - k_{y0})^2 + (k_z - k_{z0})^2} < 0.03$. This table shows only part of the full dataset, the full dataset is distributed over Tables 4-14.

Z(k_{y0}=0.07 k_{z0}=1.26)/Ω	Z(k_{y0}=0.08 k_{z0}=0.86)/Ω	Z(k_{y0}=0.27 k_{z0}=0.92)/Ω	Z(k_{y0}=0.28 k_{z0}=0.71)/Ω	Z(k_{y0}=0.9 k_{z0}=1.28)/Ω	Z(k_{y0}=1.24 k_{z0}=0.35)/Ω	Z(k_{y0}=0.71 k_{z0}=0.14)/Ω
313.6	131.5	142.8	155.1	97.8	70.1	120.6
244.3	131.7	143.3	154.2	89	74.3	121.1
224.6	131.8	138.6	153.4	83.2	74	121.5
217.3	131	138.9	152.6	78.2	73.7	122
215.4	131	139.1	151.8	73.7	73.4	122.5
218.8	131	139.4	151.1	69.9	73.1	123
226.1	131	139.8	150.4	66.7	72.8	123.6
236.3	131	140.1	149.7	63.9	72.4	124.1
248.6	131	140.5	149	61.7	72	124.7
711.9	131.1	140.8	148.3	59.9	71.6	125.3
704.6	131.2	141.2	147.7	58.5	71.2	125.8
663.9	131.2	141.7	147.1	132.5	70.8	118.9
571.2	131.3	142.1	146.5	119.5	70.3	119.3
427.1	131.5	142.6	145.9	107.8	69.9	119.8
305.8	131.6	143	154.5	97.5	73.4	120.2
240.5	131.7	139.1	153.7	89.2	73.1	120.6
223.5	131.1	139.4	152.9	83.4	72.8	121.1
216.7	131	139.7	152.1	78.3	72.4	121.6
215.3	131.1	140	151.4	73.9	72.1	122.1
219	131.1	140.3	150.6	70	71.7	122.6
226.6	131.1	140.7	149.9	66.7	71.4	123.1
237	131.2	141.1	149.2	64	71	123.7
661.2	131.2	141.5	148.6	61.7	70.5	124.2
561	131.3	141.9	147.9	59.9	70.1	124.8
416.7	131.4	142.3	147.3	119.1	69.6	119.3
297.9	131.5	142.8	146.7	107.5	72.1	119.8
236.6	131.7	139.9	153.2	97.3	71.8	120.2
222.3	131.1	140.2	152.4	89.4	71.4	120.7
216.1	131.2	140.6	151.6	83.6	71.1	121.2
215.2	131.2	140.9	150.9	78.5	70.7	121.7
219.3	131.3	141.3	150.2	74	70.3	122.2
289.7	131.4	141.7	149.5	70.1	69.9	122.7
232.7	N/A	142.1	148.8	66.8	N/A	123.2
221.2	N/A	N/A	148.2	64	N/A	120.3
215.5	N/A	N/A	N/A	89.7	N/A	120.7
N/A	N/A	N/A	N/A	83.8	N/A	N/A
N/A	N/A	N/A	N/A	78.7	N/A	N/A
N/A	N/A	N/A	N/A	74.2	N/A	N/A

[1] Y. Lu, N. Jia, L. Su, C. Owens, G. Juzeliūnas, D. I. Schuster, and J. Simon, Probing the berry curvature and fermi arcs of a weyl circuit, Phys. Rev. B **99**, 020302 (2019).

[2] J. Bao, D. Zou, W. Zhang, W. He, H. Sun, and X. Zhang, Topoelectrical circuit octupole insulator with topologically protected corner states, Physical Review B **100**, 201406 (2019).

[3] W. Zhang, D. Zou, W. He, J. Bao, Q. Pei, H. Sun, and X. Zhang, Topolectrical-circuit realization of 4d hexadecapole insulator, arXiv preprint arXiv:2001.07931 (2020).
